# Analytical Treatment Interruption after Short-Term Antiretroviral Therapy in a Postnatally Simian-Human Immunodeficiency Virus-Infected Infant Rhesus Macaque Model

**DOI:** 10.1128/mBio.01971-19

**Published:** 2019-09-05

**Authors:** Ria Goswami, Ashley N. Nelson, Joshua J. Tu, Maria Dennis, Liqi Feng, Amit Kumar, Jesse Mangold, Riley J. Mangan, Cameron Mattingly, Alan D. Curtis, Veronica Obregon-Perko, Maud Mavigner, Justin Pollara, George M. Shaw, Katharine J. Bar, Ann Chahroudi, Kristina De Paris, Cliburn Chan, Koen K. A. Van Rompay, Sallie R. Permar

**Affiliations:** a Duke Human Vaccine Institute, Duke University Medical Center, Durham, North Carolina, USA; b Duke Clinical Research Institute, Duke University Medical Center, Durham, North Carolina, USA; c Department of Pediatrics, Emory University School of Medicine, Atlanta, Georgia, USA; d Department of Microbiology and Immunology, School of Medicine, University of North Carolina at Chapel Hill, Chapel Hill, North Carolina, USA; e Center for AIDS Research, School of Medicine, University of North Carolina at Chapel Hill, Chapel Hill, North Carolina, USA; f Department of Surgery, Duke University School of Medicine, Durham, North Carolina, USA; g Department of Medicine, University of Pennsylvania, Philadelphia, Pennsylvania, USA; h Emory+Children’s Center for Childhood Infections and Vaccines, Atlanta, Georgia, USA; i Department of Biostatistics and Bioinformatics, Duke University Medical Center, Durham, North Carolina, USA; j California National Primate Research Center, University of California, Davis, California, USA; k Department of Pediatrics, Duke University School of Medicine, Durham, North Carolina, USA; University of Pittsburgh School of Medicine; Oregon Health & Science University; Tulane National Primate Research Ctr

**Keywords:** analytical treatment interruption, HIV reservoir, pediatric HIV cure, SHIV

## Abstract

To achieve long-term viral remission in human immunodeficiency virus (HIV)-infected children, novel strategies beyond early antiretroviral therapy (ART) will be necessary. Identifying clinical predictors of the time to viral rebound upon ART interruption will streamline the development of novel therapeutic strategies and accelerate their evaluation in clinical trials. However, identification of these biomarkers is logistically challenging in infants, due to sampling limitations and the potential risks of treatment interruption. To facilitate the identification of biomarkers predicting viral rebound, we have developed an infant rhesus macaque (RM) model of oral simian-human immunodeficiency virus (SHIV) SHIV.CH505.375H.dCT challenge and analytical treatment interruption (ATI) after short-term ART. We used this model to characterize SHIV replication kinetics and virus-specific immune responses during short-term ART or after ATI and demonstrated plasma viral rebound in 5 out of 6 (83%) infants. We observed a decline in humoral immune responses and partial dampening of systemic immune activation upon initiation of ART in these infants. Furthermore, we monitored SHIV replication and rebound kinetics in infant and adult RMs and found that both infants and adults demonstrated equally potent virus-specific humoral immune responses. Finally, we validated our models by confirming a well-established correlate of the time to viral rebound, namely, the pre-ART plasma viral load, as well as identified additional potential humoral immune correlates. Thus, this model of infant ART and viral rebound can be used and further optimized to define biomarkers of viral rebound following long-term ART as well as to preclinically assess novel therapies to achieve a pediatric HIV functional cure.

## Abstract (precis)

Novel interventions that do not rely on daily adherence to ART are needed to achieve sustained viral remission for perinatally infected children, who currently rely on lifelong ART. Considering the risks and expense associated with ART interruption trials, the identification of biomarkers of viral rebound will prioritize promising therapeutic intervention strategies, including anti-HIV Env protein therapeutics. However, comprehensive studies to identify those biomarkers are logistically challenging in human infants, demanding the need for relevant nonhuman primate models of HIV rebound. In this study, we developed an infant RM model of oral infection with simian-human immunodeficiency virus expressing clade C HIV Env and short-term ART followed by ATI, longitudinally characterizing the immune responses to viral infection during ART and after ATI. Additionally, we compared this infant RM model to an analogous adult RM rebound model and identified virologic and immunologic correlates of the time to viral rebound after ATI.

## INTRODUCTION

Despite the widespread availability and effectiveness of antiretroviral therapy (ART), each year >180,000 infants continue to become infected with human immunodeficiency virus (HIV) (1). Acquiring HIV at this early age commits these children to life-long ART, since stopping therapy is universally associated with viral rebound. However, continuous access to ART can be challenging in resource-limited settings (2), leading to treatment interruption and poor clinical outcomes. Maintaining adherence to lifelong therapy is particularly challenging among adolescents (3), resulting in the development of drug-resistant viral strains (4). Even if adherence is maintained, chronic exposure to ART from a young age predisposes children to drug-associated metabolic complications (5). Therefore, novel intervention strategies that do not rely on daily ART will be needed for sustained viral remission in infected children. While the establishment of viral reservoirs may not be prevented even when ART is initiated within hours of HIV infection (6), a reduced size of the latent reservoir has been demonstrated to lengthen the time to viral rebound in clinical trials (7–9). Therefore, reducing the size of the viral reservoir and attaining sustained viral remission after treatment discontinuation have been the focus of an emerging global effort aimed at developing a cure for HIV infection.

As new therapeutic interventions to attain drug-free viral remission are developed and assessed in clinical trials, safe means to measure their efficacy will be needed. While mathematical models to predict the viral rebound time from the reservoir size have been developed (10–12), this approach is limited by the inaccuracy of existing assays to measure the viral reservoir size (13) and interpatient variability in the response to identical treatment strategies. Therefore, careful monitoring of viral rebound after analytical treatment interruption (ATI) still remains the “gold standard” for the accurate validation of the efficacy of any novel anti-HIV therapeutic strategy. However, this approach is logistically challenging and carries considerable risk of virus transmission and replenishment of the viral reservoir upon reactivation. More importantly, this strategy is ethically challenging in HIV-infected children, since the outcomes of ATI studies on long-term pediatric health are not known. Considering these risks, the identification of biomarkers to serve as predictors of the time to HIV rebound (14) would be useful to prioritize the development of treatment strategies, avoiding the cost and risk of ATI studies that are unlikely to have clinical efficacy.

Virologic and immunologic biomarkers predicting HIV rebound have been identified by several studies in recent years (15–18), yet our understanding of the predictors of HIV rebound in the setting of maturing infant immune systems is limited. These types of comprehensive studies are further complicated in infants due to the limited volumes of samples that can be collected at this age. Thus, pediatric rhesus macaque (RM) models of HIV infection and treatment can be instrumental (19). Building on pediatric RM models of breast milk transmission (20) and persistence (21) with simian immunodeficiency viruses (SIVs), here we have developed a pediatric RM model of ART and viral rebound using infant RMs experimentally infected with a next-generation chimeric simian-human immunodeficiency virus (SHIV), SHIV.CH505.375H.dCT (22), that will permit an assessment of interventions directed against HIV Env. This virus carries a mutation in the CD4 binding site that facilitates entry via the rhesus macaque CD4 molecule and that has previously been demonstrated to replicate efficiently in adult RMs (22), recapitulating the viral replication dynamics and immunopathogenesis of HIV infection in humans (23). We used the infant RM model to characterize the replication kinetics and virus-specific humoral immune responses during short-term ART and after ATI. We also utilized a unique opportunity to compare the viral and immune response kinetics of infant monkeys to that of adults infected with the same virus during ART and after ATI. Furthermore, we validated and assessed these RM models by examining clinically established biomarkers of the time to viral rebound and explored the relationship between the immune response and viral rebound. This infant RM ATI model will be a valuable addition to the HIV cure research toolbox to guide translational studies for evaluating the efficacy of therapeutic strategies toward attaining drug-free HIV remission for children.

## RESULTS

### Kinetics of SHIV.CH505.375H.dCT replication in orally infected infant RMs.

**FIG 1:**
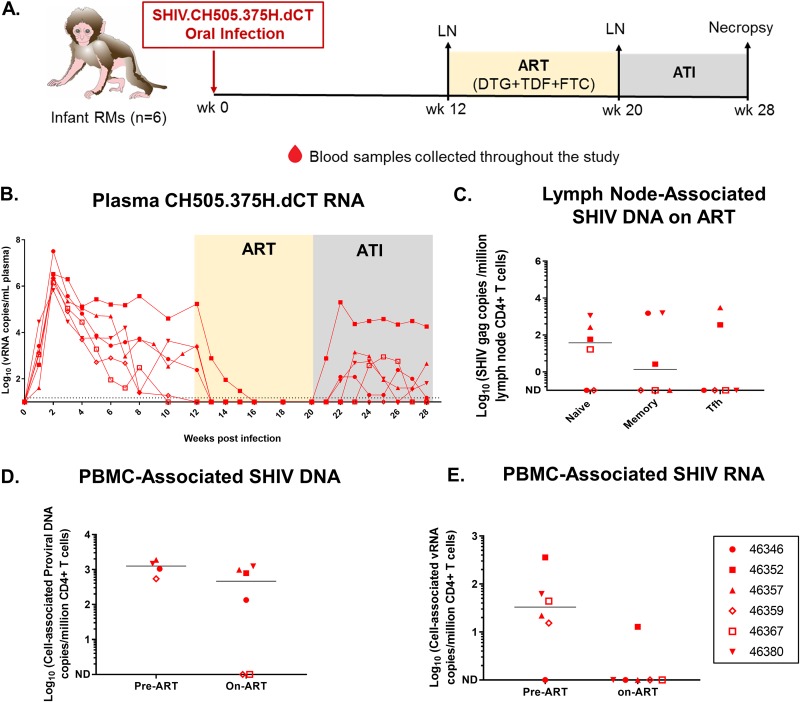
SHIV.CH505.375H.dCT replication kinetics prior to and following ATI in infant RMs. (A) Schematic representation of SHIV.CH505.375H.dCT infection (0 to 12 weeks), ART (12 to 20 weeks), and ATI (20 to 28 weeks) in infant RMs. Blood samples were collected at weekly intervals throughout the study, and peripheral lymph nodes (LNs) were collected at 12 wpi and while the RMs were on ART (20 wpi), (B) The kinetics of plasma SHIV RNA over 28 weeks were measured by qRT-PCR. (C) Peripheral lymph nodes from RMs on ART (20 wpi) were collected, and the level of naive, memory, and Tfh CD4^+^ T cell-associated SHIV DNA was estimated by qPCR. (D and E) The amounts of cell-associated SHIV DNA (CA-SHIV DNA) (D) and cell-associated SHIV RNA (CA-SHIV RNA) (E) per million CD4^+^ T cells in peripheral blood were monitored by ddPCR in the infant RMs before ART (6 wpi) and on ART (18 wpi). The sensitivity of the ddPCR assay was detection of 1 SHIV *gag* copy in 10,000 CD4^+^ T cells. Therefore, only those animals that had ≥10,000 CD4^+^ T cells at a particular time point were included in the analysis. Each symbol represents an individual animal. Yellow and gray boxes represent the duration of ART (weeks 12 to 20) and the duration of ATI (weeks 20 to 28), respectively. Medians are indicated as horizontal lines on the dot plots. Infants with a plasma VL of <15 copies/ml at 12 wpi are represented by open symbols.

Six infant RMs were orally challenged with SHIV.CH505.375H.dCT (22) by bottle feeding 3 times/day for 5 days at a dose of 8.5 × 10^4^ 50% tissue culture infective doses (TCID_50_) to mimic breast milk transmission. After a week of challenge, only 1 infant became infected, which is not surprising, considering the low rate of natural transmission in macaques during breast-feeding (24, 25). To have better control over the challenge dosage and the timing of infection, the remaining 5 RMs were sedated and orally challenged weekly at a dose of 6.8 × 10^5^ TCID_50_. After 3 weeks, one infant remained uninfected and was subsequently challenged with increasing doses (1.3 × 10^6^ TCID_50_, followed by 3.4 × 10^6^ TCID_50_) until it became infected (Table 1). The kinetics of SHIV replication in these infants were monitored for 12 weeks postinfection (wpi), when they were initiated on a daily subcutaneous ART regimen of tenofovir disoproxil fumarate (TDF), emtricitabine (FTC), and dolutegravir (DTG) for 8 weeks. After 8 weeks of ART, treatment was interrupted and the infants were monitored for an additional 8, weeks followed by necropsy (Fig. 1A).

**TABLE 1:** SHIV.CH505.375H.dCT-infected rhesus macaques, weeks of challenges to infection, age at infection, sex, time to viral control post-ART, and time to viral rebound post-ATI

Animal group	Animal identifier	No. of challenges to infection (wk)	Age at infection^*a*^	Sex^*b*^	Pre-ART control^*c*^^,^^*e*^	Time to viral control post-ART (wk)^*f*^	Viral rebound post-ATI^*c*^^,^^*g*^	Time to viral rebound post-ATI (wk)	Postrebound control^*c*^^,^^*d*^,^*h*^
Infant	46357	1	5	M	N	1	Y	3	N
	46346	2	9	F	N	1	Y	6	Y
	46352	2	9	F	N	4	Y	1	N
	46359	3	10	F	Y	NA^*d*^	N	NA	NA
	46367	7	14	M	Y	NA	Y	4	Y
	46380	4	11	F	N	1	Y	3	N
Adult	39472	1	8	F	N	1	N	NA	NA
	43068	1	5	F	N	1	N	NA	NA
	43268	1	4	F	N	1	Y	3	N
	42368	1	5	F	N	1	Y	3	Y
	39950	1	8	F	N	4	Y	2	N
	38200	1	10	F	N	1	N	NA	NA

a Ages are in weeks for infants and years for adults.

b M, male; F, female.

c N, no; Y, yes.

d NA, not applicable.

e Pre-ART control, plasma VL of ≤15 copies/ml at ART start (12 wpi).

f Viral control post-ART, plasma VL of ≤15 copies/ml after ART start.

g Viral rebound, plasma VL of ≥10 times the detection limit of the assay (150 copies of vRNA/ml plasma) post-ART discontinuation.

h Postrebound control, plasma VL of ≤15 copies/ml after 8 weeks post-ATI in monkeys showing viral rebound.

In the acute phase of infection, the plasma viral load (VL) peaked at 2 wpi (6.7 × 10^5^ to 3.2 × 10^7^ viral RNA [vRNA] copies/ml of plasma) and then declined over time (Fig. 1B). Most of the RMs did not achieve a stable VL set point, and 2 had plasma VLs less than the limit of detection (LOD) of 15 copies/ml before ART initiation (Table 1). One of these 2 infants was most resistant to infection (Table 1), and neither infant had a major histocompatibility complex (MHC) allele that has been previously associated with SHIV control (26, 27) (see Table S1 in the supplemental material). Of note, these RMs were genotyped for only a restricted set of MHC alleles that are routinely tested at the primate center to recruit animals into SHIV infection studies. Therefore, the possibility that these RMs were positive for other MHC alleles associated with spontaneous SIV control, such as B*08 (28) and B*17 (29), cannot be completely ruled out. The CD4^+^ T cell frequencies of the RMs were generally stable, with a slight decrease in the median frequency occurring at between 2 and 3 wpi (Fig. S1A), similar to the transient peripheral CD4^+^ T cell decline in acute HIV infection.

10.1128/mBio.01971-19.1 FIG S1 Proportions of CD4^+^ T cells in blood, lymph nodes, and gut-associated tissues of SHIV.CH505.375H.dCT-infected infants and adult RMs. (A) Frequencies of PBMC CD4^+^ T cells in SHIV.CH505.375H.dCT-infected infant and adult RMs through 28 wpi and 32 wpi, respectively. (B) Proportions of CD4^+^ T cells of CD3^+^ T cells in PBMCs, oral and gut-associated lymphoid tissues, and spleens of infected infant RMs at necropsy (28 wpi) and adult RMs at necropsy (32 wpi). Red symbols represent infants, and blue symbols represent adults. Each symbol represents one animal. Yellow and gray boxes represent the duration of ART and the duration of ATI, respectively. Infants with a plasma VL of <15 copies/ml at 12 wpi are represented by open symbols. Download FIG S1, TIF file, 1.8 MB. Copyright © 2019 Goswami et al. 2019 Goswami et al. This is an open-access article distributed under the terms of the Creative Commons Attribution 4.0 International license.

10.1128/mBio.01971-19.6 TABLE S1 MHC class I genotype of infant and adult RMs. Download Table S1, DOCX file, 0.01 MB. Copyright © 2019 Goswami et al. 2019 Goswami et al. This is an open-access article distributed under the terms of the Creative Commons Attribution 4.0 International license.

Upon ART initiation, the infant RMs demonstrated plasma VLs less than the LOD within 1 to 4 weeks, and none of them had detectable plasma VLs during the short course of ART. Upon ATI, 5 of 6 infants had viral rebound within 1 to 6 weeks (median, 3 weeks), and 2 of these 5 infants demonstrated plasma VLs less than the LOD within 2 to 3 weeks of viral rebound (Table 1). Interestingly, 1 of the 2 animals with VLs of <15 copies prior to ART (animal 46367) experienced viral rebound post-ATI (Fig. 1B). Not surprisingly, the animal with persistently high pre-ART viremia (animal 46352) was the first to rebound post-ATI and experienced the highest rebound viremia.

### The SHIV.CH505.375H.dCT reservoir in peripheral LNs and PBMCs of infant RMs.

We assessed the size of the viral reservoirs of infant RMs while they were virologically controlled on ART. SHIV DNA was quantified in peripheral lymph node (LN)-associated naive, memory, and T follicular helper (Tfh) CD4^+^ T cells after 8 weeks of ART. Viral DNA was detected in all three CD4^+^ T cell populations in a subset of animals, with 4 of 6 monkeys having detectable DNA in naive CD4^+^ T cells, 3 of 6 monkeys having detectable DNA in memory CD4^+^ T cells, and 2 of 6 monkeys having detectable DNA in Tfh cells (Fig. 1C). Finally, we measured the amount of cell-associated SHIV (CA-SHIV) DNA and CA-SHIV RNA per million CD4^+^ T cells isolated from peripheral blood mononuclear cells (PBMCs) using digital droplet PCR (ddPCR). Of note, we can report CA-SHIV DNA and CA-SHIV RNA data only for those animals which had input cell counts greater than the threshold cell count for the assay (see Materials and Methods). Our data demonstrated a decrease in the amount of CA-SHIV DNA (Fig. 1D) and CA-SHIV RNA (Fig. 1E) per million CD4^+^ T cells, with only one infant (animal 46352) having detectable CA-SHIV RNA after 6 weeks of ART.

### Anatomic distribution of SHIV.CH505.375H.dCT after rebound in infant RMs.

**FIG 2:**
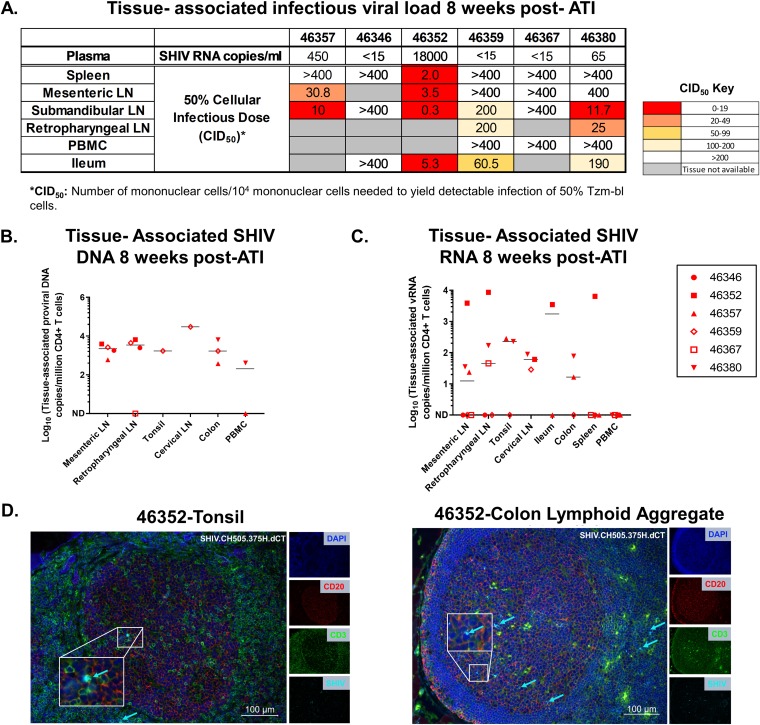
Tissue-associated infectious viral loads upon ATI in mononuclear cells isolated from PBMCs and lymphoid and gastrointestinal tissues of orally infected infant RMs. (A) Tissue-associated infectious SHIV.CH505.375H.dCT titers measured through tissue mononuclear cell coculture with TZM-bl reporter cells. The reported titers represent the estimated minimum number of mononuclear cells per 10^4^ mononuclear cells required to yield detectable infection of 50% of TZM-bl cells (CID_50_). (B and C) CD4^+^ T cell-associated proviral DNA (B) and CD4^+^ T cell-associated viral RNA (C) loads at necropsy (28 wpi), reported as the copy number per million CD4^+^ T cells in PBMCs and lymphoid and gastrointestinal tissue mononuclear cells. Each symbol represents one individual animal. Medians are indicated as horizontal lines on the dot plots. Infants with a plasma VL of <15 copies/ml at 12 wpi are represented by open symbols. The sensitivity of the ddPCR assay was detection of 1 SHIV *gag* copy in 10,000 CD4^+^ T cells. Therefore, only those animals that had ≥10,000 CD4^+^ T cells at a particular time point were included in the analysis. (D) Tonsil and colon sections from the SHIV.CH505.375H.dCT-infected infant RM that demonstrated the highest peak plasma VL postrebound (20,000 vRNA copies/ml plasma). Tissue sections were stained with the nuclear marker DAPI (4′,6-diamidino-2-phenylindole; dark blue) to identify cells and with antibodies specific for CD3 (green) and CD20 (red). Virus-infected cells were identified by *in situ* hybridization (cyan). To better visualize the virus-infected cells, we magnified a specific region (white box) in each image. Each panel consists of a larger image with the overlay of all markers and 4 smaller side panels of the same field for each individual channel. Arrow colors correspond to the color for the indicated marker. The large image has a scale bar in the lower right corner.

As anatomic sites of viral replication after ATI might reveal major sources of viral rebound, we sought to determine the distribution of SHIV.CH505.375H.dCT in blood and tissue compartments at necropsy. Cell-associated infectious SHIV was measured in oral and gut-associated tissues (at 8 weeks post-ATI) using a TZM-bl cell-based coculture assay (Fig. S2), and the 50% cellular infectious dose (CID_50_) for each tissue was reported (see Materials and Methods). Our data demonstrated that the infectious virus was primarily distributed in the LNs and gut-associated tissues rather than the spleen (Fig. 2A), which might be attributed to the lower proportion of CD4^+^ T cells in the spleen (Fig. S1B) than in LNs. Interestingly, a higher number of animals had infectious virus detectable in the oral LN (submandibular LN) than in the mesenteric LN, and no cell-associated infectious virus was detected in PBMCs.

10.1128/mBio.01971-19.2 FIG S2 Tissue-associated infectious viral levels in RMs infected with SHIV.CH505.375H.dCT. Mononuclear cells isolated from the tissues of infant (A) and adult (B) RMs infected with SHIV.CH505.375H.dCT were serially diluted and cocultured with TZM-bl reporter cells for 72 h, followed by luminescent detection of tissue-associated SHIV infectivity in relative luminescence units (RLU). The RLU limit of detection for positive tissue-associated SHIV infection (dashed line) was defined as 2.5 times the mean maximum RLU elicited from TZM-bl cells (*n* = 10 independent assays) in the coculture assay. Download FIG S2, TIF file, 1.9 MB. Copyright © 2019 Goswami et al. 2019 Goswami et al. This is an open-access article distributed under the terms of the Creative Commons Attribution 4.0 International license.

At 8 weeks post-ATI, tissue-associated SHIV DNA and RNA were detectable at variable levels per million CD4^+^ T cells (SHIV DNA, 1 × 10^4^ to 3 × 10^4^ copies/million CD4^+^ T cells; SHIV RNA, 1 × 10^3^ to 8.61 × 10^3^ copies/million CD4^+^ T cells) (Fig. 2B and C). None of the monkeys had detectable SHIV RNA in PBMCs, further confirming our coculture-based tissue-associated infectious viral load data. We further defined the anatomic distribution of CD3^+^ SHIV-positive (SHIV^+^) cells in LNs and gut-associated lymphoid tissues (GALT) of the infant that showed the highest plasma VL postrebound, using a dual immunohistochemistry (IHC)/*in situ* hybridization (ISH) approach (Fig. 2D). Interestingly, CD3^+^ SHIV^+^ cells were detected within the B cell follicles, in addition to the T cell zone, suggesting that resident Tfh cells in both the tonsil and GALT can support viral replication.

### SHIV.CH505.375H.dCT replication kinetics, viral reservoir, and rebound virus distribution in adult RMs.

**FIG 3:**
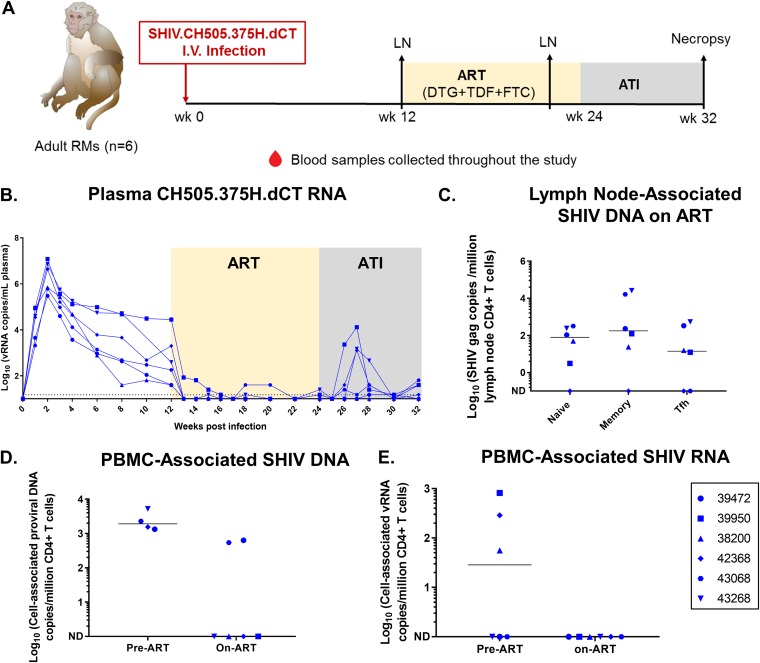
SHIV.CH505.375H.dCT replication kinetics prior to and following ATI in adult RMs. (A) Schematic representation of SHIV.CH505.375H.dCT infection (0 to 12 weeks), ART (12 to 24 weeks), and ATI (24 to 32 weeks) in adult RMs. Blood samples were collected at weekly intervals throughout the study, and peripheral lymph nodes (LNs) were collected at 12 wpi and after 8 weeks of ART (20 wpi). I.V., intravenous. (B) The kinetics of plasma SHIV RNA over 32 weeks were measured by qRT-PCR. (C) Peripheral lymph nodes from macaques on ART (20 wpi) were collected, and the level of naive, memory, and Tfh CD4^+^ T cell-associated SHIV DNA was estimated by qPCR. (D and E) The amounts of cell-associated SHIV DNA (CA-SHIV DNA) (D) and cell-associated SHIV RNA (CA-SHIV RNA) (E) from CD4^+^ T cells of peripheral blood before ART (6 wpi for DNA and 12 wpi for RNA) and on ART (18 wpi) were monitored by ddPCR. The sensitivity of the ddPCR assay was detection of 1 SHIV *gag* copy in 10,000 CD4^+^ T cells. Therefore, only those animals that had ≥10,000 CD4^+^ T cells at a particular time point were included in the analysis. Each symbol represents an individual animal. Yellow and gray boxes represent the duration of ART (weeks 12 to 24) and the duration of ATI (weeks 24 to 32), respectively. Medians are indicated as horizontal lines on the dot plots.

We took the opportunity to compare the viral replication kinetics and reservoir in infant RMs to those in adult RMs from a separate study infected with the same SHIV strain, which was thus a cohort of convenience (30). Six adult RMs were intravenously infected with SHIV.CH505.375H.dCT (see Materials and Methods) and started on a triple-ART regimen of TDF, FTC, and DTG at 12 wpi. After 12 weeks of ART, therapy was discontinued, and the animals were euthanized at 8 weeks post-ATI (Fig. 3A). As in the infant RMs, the plasma VL in adults peaked at 2 wpi (3 × 10^5^ to 1.2 × 10^7^ copies of vRNA/ml of plasma). Additionally, the overall kinetics of the plasma VL during acute SHIV infection were highly comparable between the two groups (Fig. 3B). Upon ART initiation, the plasma VL in adults was below the LOD (<15 copies/ml plasma) within 1 to 4 weeks, with one monkey experiencing a viral blip (>15 copies/ml plasma) during the course of ART. Even though the ART regimen in the adults was slightly longer than that in the infants, 3 of 6 adults showed viral rebound within 2 to 3 weeks post-ATI. Interestingly, 1 of 3 adults that experienced viral rebound demonstrated a plasma VL below the LOD at 8 weeks post-ATI (Table 1). A correlation trend was observed between the pre-ART plasma VL and peak acute rebound VL (Fig. S3), with no significant difference in the correlation (Kendall’s tau value) being seen between the two age groups (mean tau difference = 0.258; *P* = 0.656). This indicates that a higher seeding of the viral reservoir before treatment might contribute to higher post-ATI viremia in both age groups.

10.1128/mBio.01971-19.3 FIG S3 Correlation of plasma viral load at 12 wpi (pre-ART) with the peak acute rebound viremia. The correlations between the plasma VL at 12 wpi and the peak acute rebound plasma VL in infant RMs (A), adult RMs (B), and both age groups combined (C) are shown. The coefficients of correlation (Kendall’s tau values) and *P* values from testing whether the correlation coefficients differed significantly from 0 are shown on the graphs. Download FIG S3, TIF file, 1.5 MB. Copyright © 2019 Goswami et al. 2019 Goswami et al. This is an open-access article distributed under the terms of the Creative Commons Attribution 4.0 International license.

**FIG 4:**
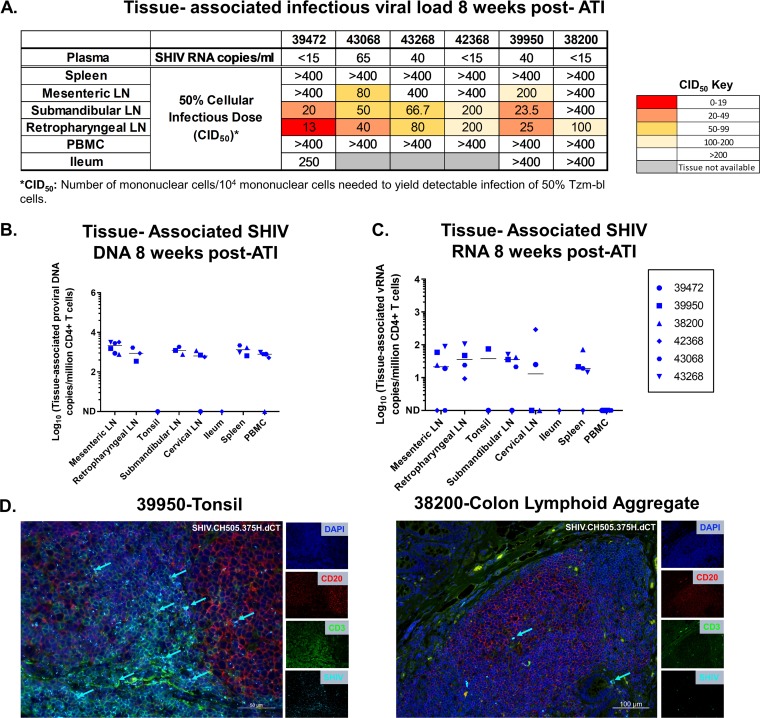
Tissue-associated infectious virus load upon ATI in mononuclear cells isolated from PBMCs and lymphoid and gastrointestinal tissues of adult RMs intravenously infected with SHIV.CH505.375H.dCT. (A) Tissue-associated infectious SHIV.CH505.375H.dCT titers measured through tissue mononuclear cell coculture with TZM-bl reporter cells. The reported titers represent the estimated minimum number of mononuclear cells per 10^4^ mononuclear cells required to yield detectable infection of 50% of TZM-bl cells (CID_50_). (B and C) CD4^+^ T cell-associated proviral DNA (B) and viral RNA loads (C), reported as the copy number per million CD4^+^ T cells in PBMCs and lymphoid and gastrointestinal tissue mononuclear cells. Each symbol represents one individual monkey at necropsy (week 32 postinfection). Medians are indicated as horizontal lines on the dot plots. (D) Tonsil and colon sections from the SHIV.CH505.375H.dCT-infected adult RM (animal 39950) that demonstrated the highest peak plasma VL postrebound (13,000 vRNA copies/ml plasma). Tissue sections were stained with the nuclear marker DAPI (dark blue) to identify cells and with antibodies specific for CD3 (green) and CD20 (red). Virus-infected cells were identified by *in situ* hybridization (cyan). Each panel consists of a larger image with the overlay of all markers and 4 smaller side panels of the same field for each individual channel. Arrow colors correspond to the color for the indicated marker. The large image has a scale bar in the lower right corner.

The SHIV reservoir was detectable in the naive, memory, and Tfh CD4^+^ T cell subsets of the peripheral LNs of the adult RMs (Fig. 3C). As was observed with the infant RMs, the levels of CA-SHIV DNA (Fig. 3D) and CA-SHIV RNA (Fig. 3E) per million CD4^+^ T cells declined upon ART, with only 2 adults demonstrating detectable CA-SHIV DNA levels after 6 weeks on ART. Like infant RMs, coculture assays detected higher infectious viral titers in the oral LN (submandibular LN) than in the mesenteric LN, whereas cell-associated infectious virus was not detected in PBMCs (Fig. 4A). CA-SHIV DNA and RNA were detected in tissues at variable levels, with none of the animals having detectable CA-SHIV RNA in PBMCs (Fig. 4B and C). Similar to infants, the tonsils and colon lymphoid aggregates of the adult RMs that showed the highest plasma VL postrebound had detectable CD3^+^ SHIV^+^ cells within the B cell follicle, in addition to the T cell zone, at necropsy (Fig. 4D).

### Viral diversity before ART initiation and after ATI in infant and adult RMs.

**FIG 5:**
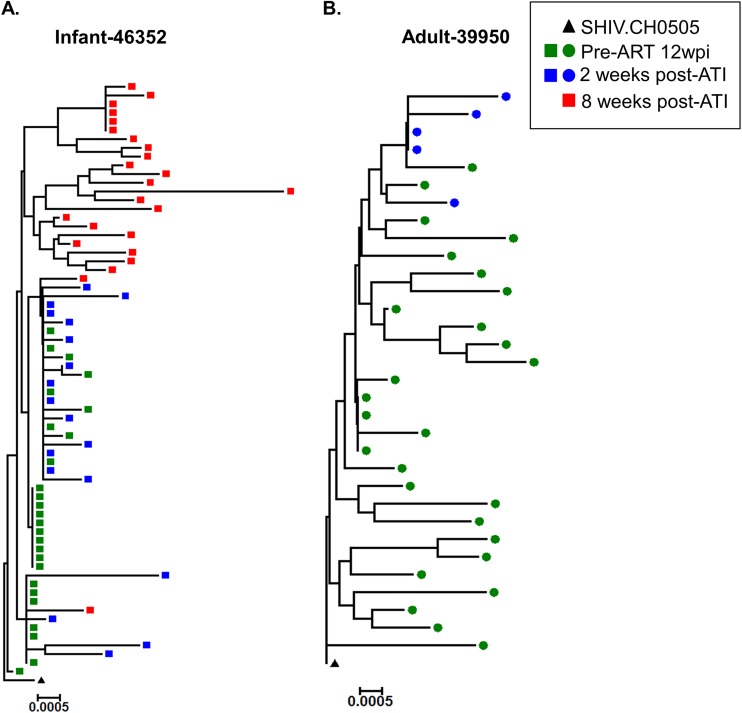
Phylogenetic tree analysis of the *env* gene sequences obtained pre-ART and post-ATI from the plasma of infant and adult RMs demonstrating the highest peak plasma VL postrebound. Standard SGA techniques were used to analyze the *env* gene from pre-ART and post-ATI samples from infants and adult RMs with the highest peak rebound plasma VL. The phylogenetic tree represents the viral *env* diversity in infant 46352 (A) and adult 39950 (B).

We next sought to compare the plasma viral Env diversity between the infant RM (animal 46352) and the adult RM (animal 39950) that showed the highest plasma VL postrebound. We performed single-genome amplification (SGA) and sequencing of the *env* gene collected from plasma pre-ART and post-ATI and calculated the average pairwise distance (APD) within Env sequences. In the infant (animal 46352), the viral Env pre-ART (12 wpi) was more homogeneous (APD, 0.0006) than the viral Env at 2 weeks (APD, 0.0018) or 8 weeks (APD, 0.0038) post-ATI (Fig. 5A). In contrast, for the adult RM (animal 39950), the viral Env pre-ART (12 wpi) had a 4-fold higher diversity (APD, 0.004) than it did at 2 weeks post-ATI (APD, 0.001) (Fig. 5B).

### ART dampens the magnitude of humoral responses in SHIV-infected infant and adult RMs.

**FIG 6:**
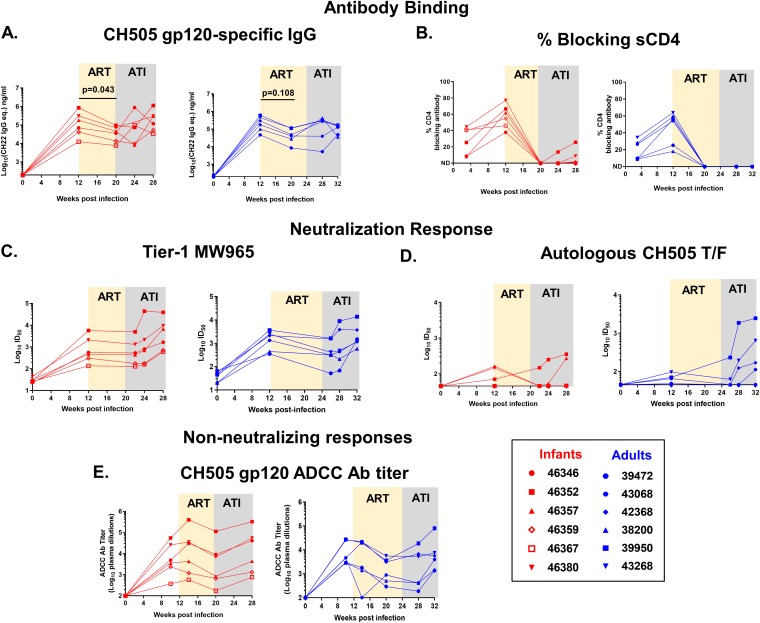
Magnitude and kinetics of humoral responses to acute SHIV.CH505.375H.dCT infection during ART and ATI in RMs. Plasma from infant and adult rhesus macaques was analyzed for the HIV CH505 gp120 IgG response (A), blocking of soluble CD4-gp120 interactions (B), the tier 1 neutralization response against MW965 (C), the neutralization response against the CH505 T/F virus (D), and the ADCC titer against CH505 gp120-coated target cells (E). Red symbols represent infant RMs, and blue symbols represent adult RMs. Each symbol represents an individual macaque. Yellow and gray boxes represent the duration of ART and the duration of ATI in the RMs, respectively. Infants with a plasma VL of <15 copies/ml at 12 wpi are represented with open symbols. *P* values were calculated using the Wilcoxon signed-rank test. eq., equivalents; Ab, antibody; ID_50_, 50% infective dose.

As previous reports have noted a loss of HIV-specific humoral responses in human infants on ART (31), we investigated the differences in the kinetics, magnitude, and breadth of the Env-specific humoral responses between the age groups on ART and following ATI. In both age groups, all monkeys developed detectable autologous gp120-specfic IgG responses at 12 wpi (Fig. 6A). After 8 weeks of ART, gp120-specific IgG response declined in both groups, with the decline being more pronounced in infants, yet the gp120-specific IgG response rebounded in both groups post-ATI. Interestingly, the infant (animal 46359) with a plasma VL of <15 copies pre-ART and no viral rebound developed a gp120-specific IgG response similar to that of the other infants. We then mapped the Env domain specificity of the antibody responses and observed dominant responses against the V3 and C5 linear epitopes in both groups (Fig. S4), which were not altered upon ART. Interestingly, ART initiation completely abrogated the plasma antibody response against the CD4 binding site, and only 2 of 6 infants and no adults regained this response within the 8 weeks of follow-up after ART was discontinued (Fig. 6B).

10.1128/mBio.01971-19.4 FIG S4 Specificity of Env IgG responses before and on ART in SHIV.CH505.375H.dCT-infected RMs. The plasma IgG specificity against a panel of HIV Env linear and conformational epitopes pre-ART (12 wpi) and on ART (20 wpi) in SHIV.C.CH505-infected infant (A) and adult (B) RMs is shown. Heat maps represent the mean fluorescence intensity (MFI) of IgG binding to each epitope. Download FIG S4, TIF file, 1.7 MB. Copyright © 2019 Goswami et al. 2019 Goswami et al. This is an open-access article distributed under the terms of the Creative Commons Attribution 4.0 International license.

We next evaluated the HIV neutralization potency of RM plasma against tier 1 clade-matched isolate MW965 and the autologous tier 2 CH505 virus. Both infants and adults developed neutralization activity against MW965 by 12 wpi, which continued to increase post-ATI, with the potency being equal between the age groups (Fig. 6C). However, only half of both the adult and the infant RMs had a detectable neutralization response against autologous CH505 at 12 wpi (Fig. 6D). Post-ATI, 2 of 6 infants and 4 of 6 adults demonstrated increasing neutralization responses against the autologous virus. In both age groups, gp120-specific antibody-dependent cellular cytotoxicity (ADCC) titers dampened upon ART initiation, yet they recovered to pre-ART levels after ATI (Fig. 6E).

### T cell activation during ART in infant and adult RMs.

**FIG 7:**
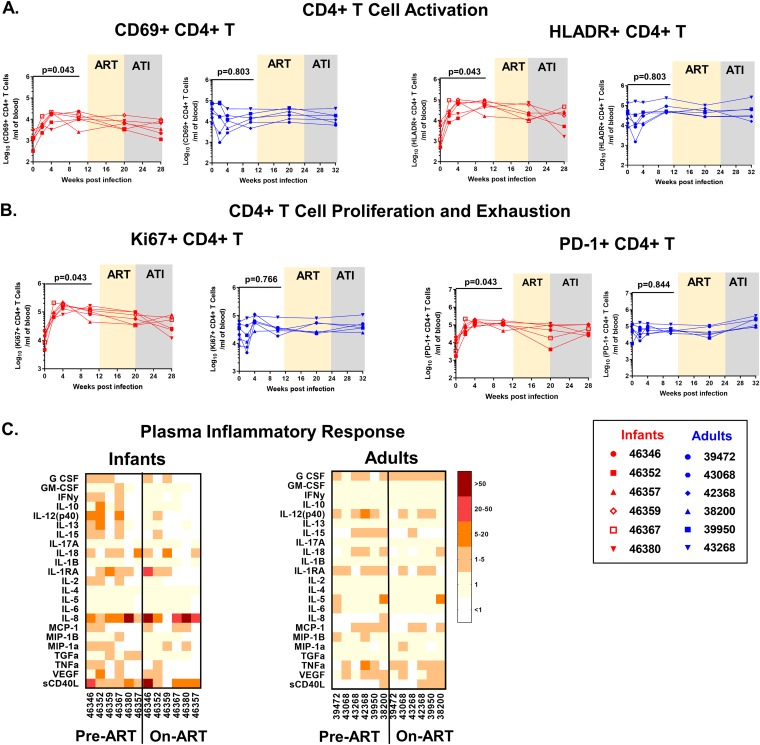
CD4^+^ T cell activation and plasma inflammatory responses in SHIV.CH505.375H.dCT-infected infant and adult RMs. Absolute counts per milliliter of blood of activated (CD69^+^ HLA-DR^+^) CD4^+^ T cells (A) and proliferating (Ki67^+^ CD4^+^) and exhausted (PD-1^+^) CD4^+^ T cells (B). Red symbols represent infant RMs, and blue symbols represent adult RMs. Each symbol represents one animal. Yellow and gray boxes represent the duration of ART and the duration of ATI, respectively. (C) Plasma from infected infant and adult RMs was analyzed by a multiplexed Luminex assay for expression of cytokines before ART (week 12) and after 8 weeks on ART (week 20). The heat maps represent the fold change in the level of each analyte over preinfection plasma levels. Infants with a plasma VL of <15 copies/ml at 12 wpi are represented with open symbols. *P* values were calculated using the Wilcoxon signed-rank test.

Systemic immune activation has been associated with HIV replication and poor disease outcomes (32). Furthermore, T cell exhaustion markers have been reported to be predictors of viral rebound in a human study (15). Therefore, we assessed the activation and exhaustion status of T cells from SHIV-infected infant and adult RMs. Unlike the populations from adults, infant activated, proliferating, and exhausted CD4^+^ T cell population levels increased at 10 wpi compared to the preinfection levels, which might be attributed to the age-specific development of T cell populations (Fig. 7A and B).

Furthermore, we measured the concentrations of inflammatory chemokines and cytokines in RM plasma before ART (12 wpi) and while the animals were on ART (8 weeks of ART). While infant plasma demonstrated a higher magnitude and breadth of cytokine and chemokine levels than adult plasma, no notable difference between the values before ART and while the animals were on ART was observed in either age group (Fig. 7C).

### Correlates of time to SHIV rebound in infant and adult RMs.

To validate our established RM SHIV rebound models in identifying correlates of the time to viral rebound, we performed univariate Cox proportional hazard modeling on a subset of the measured virologic and immunologic parameters, after adjustment for age. We defined viral rebound as plasma VL of >10 times the LOD (150 copies/ml) post-ART discontinuation. The plasma VL pre-ART, ADCC antibody titers, CD4^+^ CD69^+^ T cell counts, and autologous virus neutralizing antibody titers were preselected as primary parameters, based on their previously published associations with the rate of HIV acquisition (33, 34) and disease progression (35, 36). Of these parameters, higher levels of the plasma VL pre-ART and higher ADCC antibody titers on ART demonstrated associations with an increased risk of viral rebound (Table 2). Next, we applied the model with an additional set of virologic and immunologic parameters. The analysis identified higher levels of CH505 gp120-specific IgG responses before ART and on ART, the tier 1 MW965-neutralizing antibody titer, and the percentage of soluble CD4 (sCD4)-blocking antibodies pre-ART to be associated with a higher risk of rebound (Table 2), suggesting that our macaque models are suitable for monitoring correlates of viral rebound.

**TABLE 2:** Virologic and immunologic correlates of time to viral rebound with adjustment for age^*e*^

Variable	HR (95% CI)	*P* value	
		Unadjusted	Adjusted
**Preselected variables**			
Plasma VL at ART start^*a*^	2.2 (1.24–3.95)	**0.008**	NA
ADCC antibody titer on ART^*a*^	1.9 (1.05–3.45)	**0.034**	NA
CD4*^+^* CD69^+^ T cells on ART^*b*^	0.82 (0.26–2.56)	0.727	NA
CH505 T/F-neutralizing antibody titer at ART start^*a*^	2.1 (0.82–5.57)	0.119	NA
**Exploratory variables**			
**Virologic and humoral responses**			
Peak plasma VL^*a*^	1.39 (0.86–2.27)	0.182	0.364
CH505 gp120-specific IgG pre-ART^*a*^	4.61 (1.38–15.42)	**0.0131**	0.066
CH505 gp120-specific IgG on ART^*a*^	6.06 (1.33–27.54)	**0.0197**	0.066
Tier 1 MW965-neutralizing antibody pre-ART^*a*^	4.12 (1.42–11.96)	**0.00926**	0.066
sCD4-blocking antibodies pre-ART^*c*^	1.07 (1–1.13)	**0.0402**	0.1
**CD4^+^ T cell phenotypes and inflammatory responses**			
CD4^+^ T cells pre-ART^*b*^	0.97 (0.86–1.1)	0.66	0.81
CD4^+^ T cells on ART^*b*^	0.99 (0.9–1.09)	0.77	0.82
HLA-DR^+^ CD4^+^ T cells on ART^*b*^	1 (1–1)	0.71	0.81
Ki67^+^ CD4^+^ T cells on ART^*b*^	1 (0.98–1.05)	0.4	0.8
PD-1^+^ CD4^+^ T cells on ART^*b*^	1 (1–1)	0.32	0.8
G-CSF on ART^*b*^	1.1 (0.91–1.28)	0.36	0.8
IL-12 on ART^*d*^	1.1 (0.96–1.36)	0.13	0.8
IL-15 on ART^*d*^	0.77 (0.4–1.5)	0.45	0.8
IL-18 on ART^*d*^	0.97 (0.93–1.01)	0.15	0.8
IL-1RA on ART^*d*^	1 (0.99–1.01)	0.71	0.81
IL-8 on ART^*d*^	1 (1–1)	0.97	0.97
MCP-1 on ART^*d*^	1 (0.99–1.02)	0.34	0.8
MIP-1α on ART^*d*^	0.68 (0.3–1.57)	0.37	0.8
TNF-α on ART^*d*^	0.91 (0.79–1.04)	0.17	0.8
VEGF on ART^*d*^	0.97 (0.86–1.1)	0.64	0.81
sCD40L on ART^*d*^	1 (1–1)	0.7	0.81

a Values are expressed as log_10_.

b Values are expressed as absolute counts per nanoliter of blood.

c Values are expressed as percentages.

d Values are expressed as concentrations in picogram/milliliter (pg/mL).

e HR, hazard ratio; CI, confidence interval; NA, not applicable since multiple adjustment was not performed on preselected parameters.

Finally, we wanted to determine the differential impact of age on the correlates of viral rebound. Due to the relatively small sample size of the two age groups, we performed Kendall’s tau rank correlation of each of the experimental parameters with viral rebound. In infants, a lower plasma VL at ART start was associated with a longer time to viral rebound, and a correlation trend for a longer time to viral rebound was observed for infant RMs with lower CH505 gp120-specific IgG responses before ART and on ART. In adults, lower values of the plasma VL at ART start, the peak plasma VL, the CH505 gp120-specific IgG response pre-ART, and the tier 1 MW965-neutralizing antibody titer at ART start were associated with a longer time to rebound. Additionally, a correlation trend for a longer time to viral rebound was observed for adult monkeys with lower CH505 gp120-specific IgG responses on ART (Table 3).

**TABLE 3:** Virologic and immunologic correlates of time to viral rebound in each age group

Variable	Infants			Adults		
	Kendall's tau	*P* value		Kendall's tau	*P* value	
		Unadjusted	Adjusted		Unadjusted	Adjusted
**Preselected variables**						
Plasma VL at ART start^*a*^	−0.786	**0.032**	NA^*e*^	−0.886	**0.022**	NA
ADCC antibody titer on ART^*a*^	−0.414	0.251	NA	−0.701	0.064	NA
CD4^+^ CD69^+^ T cells on ART^*b*^	0.276	0.444	NA	−0.078	0.837	NA
CH505 T/F-neutralizing antibody titer at ART start^*a*^	−0.54	0.152	NA	−0.322	0.404	NA
**Exploratory variables**						
**Virologic and humoral responses**						
Peak plasma VL^*a*^	0	1	1	−0.856	**0.024**	**0.04**
CH505 gp120-specific IgG pre-ART^*a*^	−0.69	**0.056**	0.14	−0.856	**0.024**	**0.04**
CH505 gp120-specific IgG on ART^*a*^	−0.69	**0.056**	0.14	−0.701	0.064	0.08
Tier 1 MW965-neutralizing antibody pre-ART^*a*^	−0.552	0.126	0.158	−0.856	**0.024**	**0.04**
sCD4-blocking antibodies pre-ART^*c*^	−0.552	0.126	0.158	−0.545	0.15	0.15
**CD4^+^ T cell phenotypes and inflammatory responses**						
CD4^+^ T cells pre-ART^*b*^	0.138	0.702	0.936	0.078	0.837	0.837
CD4^+^ T cells on ART^*b*^	0	1	1	0.234	0.537	0.781
HLA-DR^+^ CD4^+^ T cells on ART^*b*^	0.276	0.444	0.936	−0.078	0.837	0.837
Ki67^+^ CD4^+^ T cells on ART^*b*^	−0.276	0.444	0.936	0.078	0.837	0.837
PD-1+ CD4^+^ T cells on ART^*b*^	0.138	0.702	0.936	0.545	0.15	0.781
G-CSF on ART^*b*^	0.357	0.33	0.936	−0.418	0.289	0.781
IL-12 on ART^*d*^	−0.357	0.33	0.936	−0.261	0.511	0.781
IL-15 on ART^*d*^	−0.138	0.702	0.936	0.322	0.404	0.781
IL-18 on ART^*d*^	0.552	0.126	0.936	0.405	0.343	0.781
IL-1RA on ART^*d*^	0.138	0.702	0.936	0.389	0.304	0.781
IL-8 on ART^*d*^	0.138	0.702	0.936	0.078	0.837	0.837
MCP-1 on ART^*d*^	−0.414	0.251	0.936	−0.234	0.537	0.781
MIP-1α on ART^*d*^	0.077	0.838	0.967	0.405	0.343	0.781
TNF-α on ART^*d*^	0.357	0.33	0.936	0.234	0.537	0.781
VEGF on ART^*d*^	−0.071	0.846	0.967	0.087	0.827	0.837
sCD40L on ART^*d*^	0	1	1	−0.234	0.537	0.781

a Values are expressed as log_10_.

b Values are expressed as absolute counts per nanoliter of blood.

c Values are expressed as percentages.

d Values are expressed as concentrations in pg/mL.

e NA, not applicable since multiple adjustment was not performed on preselected parameters.

## DISCUSSION

The identification of plasma viral RNA (35) and CD4^+^ T cell counts (37) as surrogate markers of HIV disease progression was instrumental in the development of ART for attaining improved clinical care in HIV-infected patients. As the HIV field rapidly turns toward achieving functional cure, there has been renewed interest in identifying biomarkers of viral rebound. Monitoring biomarkers will guide clinical trials with effective therapeutic candidates, minimizing the investment in those that are unlikely to result in a delay of viral rebound. However, ATI trials for the identification of biomarkers remain logistically challenging, particularly in children, necessitating the development of tractable pediatric animal models. While models of ATI in SIV-infected pediatric RMs are not new to the HIV field (38), clinically translatable RM models of ATI using SHIVs have been advancing (39). Thus, we sought to establish an oral SHIV-infected infant model of ATI with a previously validated ART regimen (21).

This study investigated the impact of ART on SHIV replication and SHIV-specific immune responses in the context of the maturing infant immune system. Employing an adult RM SHIV-infected cohort of convenience, we compared the SHIV replication kinetics in infant and adult RMs and demonstrated that infants are as equipped as adults to mount immune responses both on ART and after ATI. Furthermore, we validated our RM models of ATI by confirming previously reported virologic correlates of rebound and identified additional potential humoral immune correlates.

In this study, the infant and adult RMs were infected with a clade C SHIV variant, SHIV.CH505.375H.dCT, due to the predominance of clade C viruses in sub-Saharan Africa, where most pediatric HIV infections occur (40). In both infant and adult models, this SHIV variant achieved a peak viral load comparable to that achieved in previously described models of SIV/SHIV infection (21, 41). However unlike other SHIV variants (42–45), SHIV.CH505.375H.dCT could not establish a viral set point in most of the infant and adult monkeys (Fig. 1B and 3B). Additionally, a few monkeys from our cohort achieved natural virologic control, which was not uncommon in previously described SHIV infection models (46, 47). Moreover, our study revealed that while the levels of pre-ART plasma viremia in the age groups were fairly comparable, infants demonstrated persistent plasma VL post-ATI, in contrast to the adults.

Previous studies have claimed that infants may have immune responses to HIV infection that are impaired compared to those of adults (48). However, we previously demonstrated that vaccination of infants can induce robust HIV Env-specific IgG responses (49). Moreover, we recently reported that infant monkeys are capable of mounting durable anti-HIV humoral immune responses during acute SHIV infection, despite their maturing immune landscape (30). In this study, we observed comparable humoral immune responses between the two age groups during ART and after ATI (Fig. 6). Interestingly, a decline of HIV gp120-specific IgG responses and ADCC responses was observed on ART, without a change in the specificities of the Env domain-specific antibodies (Fig. S4). A similar observation was made in human infants, where HIV-specific antibody levels decreased as the duration of ART increased (50), suggesting that circulating HIV antigen is a major driving factor for the production of HIV Env-specific antibodies. In fact, plasma Env-specific IgG levels could provide a more comprehensive measure of viral replication in tissue sanctuaries which might not be reflected in the plasma viral load.

To identify correlates of viral rebound in infants and adults, we analyzed a comprehensive panel of 25 virologic and immunologic parameters. Our data confirmed a key, well-established clinical virologic marker, the pre-ART plasma VL, to be a correlate of viral rebound in both age groups. Additionally, a higher peak plasma VL was identified as a correlate of quicker viral rebound in adults, but not infants. Furthermore, our data also indicated a correlation trend of the pre-ART plasma VL with the peak acute rebound VL (Fig. S3), which has been demonstrated previously in an HIV Gag-based therapeutic trial, where a lower pre-ART plasma VL was independently associated with a lower post-ATI plasma VL (51). Among the immunologic parameters tested, in adults, higher gp120-specific IgG responses and higher titers of neutralizing antibodies against a tier 1 virus correlated with quicker viral rebound, whereas in infants, higher gp120-specific IgG responses but not higher titers of neutralizing antibodies against a tier 1 virus showed a correlation trend with a quicker viral rebound. There has been continued interest in considering anti-HIV gp120 responses as a screening marker for ongoing viral replication or breakthrough during suppressive ART (52). Additionally, heterologous neutralizing antibody responses at the time of treatment interruption have been associated with a reduced viral load over time (53). However, these identified humoral responses have not been previously associated with the time to viral rebound and therefore should be examined in future long-term studies. In resource-limited settings, monitoring of HIV-specific humoral responses in infants on suppressive ART might be beneficial due to the small sample volume requirement and relatively low cost and technology burden compared to those of nucleic acid-based assays or quantitative viral outgrowth assays (QVOA).

There were a few notable limitations to this pilot study. First, the adult RM model of SHIV rebound used in the study was not originally designed for a direct comparison with the infant ATI model (e.g., the infection route, infection dose, and duration of therapy were not precisely matched to those in the infant study), yet the availability of a comparable adult cohort that was infected with the same SHIV variant used to infect the infant RMs and that had viral replication kinetics similar to those in the infant RMs provided us a unique opportunity to investigate the differences in the infant immune responses during and after therapy from those in adults. We also acknowledge that differences in the challenge route and duration of ART in adult RMs might have limited our ability to directly compare the immune responses in the two age groups. Moreover, the relatively small cohort sizes likely contributed to our inability to identify some of the previously established immunologic parameters of viral rebound, such as the pretherapy levels of the T cell exhaustion markers Tim-3, Lag-3, and PD-1 (15). Hence, further validation of this model in larger pediatric RM cohorts will be required. Second, since our cohorts were subjected to a very short duration of ART (8 to 12 weeks), the measured viral reservoir may not be a reflection of the true persistent reservoir on long-term suppressive ART. Therefore, we excluded measures of viral reservoir size, which have been identified to be predictors of viral rebound in previous adult clinical trials (16–18, 54). Finally, the low cell numbers collected and the comprehensive nature of the study restricted our ability to measure longitudinal T cell functions, potentially missing T cell function-associated correlates of the viral rebound time.

In conclusion, this study validated an oral SHIV-infected pediatric infant RM model of ATI and characterized SHIV replication and the humoral immune responses during and after ATI. Larger and longer-term prospective studies will be needed to further optimize this model and identify a comprehensive set of biomarkers that can reliably predict the time to viral rebound. The development of algorithms by combining several surrogate markers of viral rebound could greatly accelerate the process of screening children as candidates for ATI trials and could be used for the development of novel therapeutics for HIV cure research. Additionally, an infant HIV rebound model will be a valuable tool to identify and evaluate the potency of novel therapeutic strategies for attaining functional cure in the context of the maturing infant immune system.

## MATERIALS AND METHODS

### Animal care and study design.

Type D retrovirus-, SIV-, and simian T cell leukemia virus type 1-free Indian rhesus macaques (RM; Macaca mulatta) were maintained in the colony of the California National Primate Research Center (CNPRC; Davis, CA) as previously described (55). The CNPRC is accredited by the Association for Assessment and Accreditation of Laboratory Animal Care International (AAALAC). Animal care was performed in compliance with the 2011 Guide for the Care and Use of Laboratory Animals provided by the Institute for Laboratory Animal Research. The study was approved by the Institutional Animal Care and Use Committee of the University of California, Davis. Six infant RMs were orally challenged with SHIV.CH505.375H.dCT (22) as described previously (30). Briefly, the infant RMs were challenged at 4 weeks of age by bottle feeding 3 times/day for 5 days at a dose of 8.5 × 10^4^ TCID_50_, to mimic breast milk transmission. After 1 week of challenges, 1 infant became infected. The remaining 5 were sedated and orally challenged weekly at a dose of 6.8 × 10^5^ TCID_50_. Within 3 weeks, 4 more became infected, and the final one was challenged at increasing doses (1.3 × 10^6^ TCID_50_, followed by 3.4 × 10^6^ TCID_50_) until it became infected at 14 weeks of age (Table 1). Except for increasing the challenge dose, the conditions of inoculation were not altered between the challenges, and no noticeable differences in the phenotype or immune parameters were observed in those RMs that required multiple challenges to get infected. Six adult RMs (age range, 4 to 10 years) were intravenously infected with SHIV.CH505.375H.dCT at a dose of 3.4 × 10^5^ TCID_50_ as described previously (30). The plasma viral RNA load of the monkeys was assessed by a highly sensitive quantitative reverse transcription PCR (qRT-PCR) assay (56). A coformulation containing 5.1 mg/kg of body weight tenofovir disoproxil fumarate (TDF), 40 mg/kg emtricitabine (FTC), and 2.5 mg/kg dolutegravir (DTG) was prepared as described previously (57) and administered once daily by the subcutaneous route starting at 8 weeks (infants) or 12 weeks (adults) postinfection.

### Collection and processing of blood and tissue specimens and MHC typing of animals.

Animals were sedated with ketamine HCl (Parke-Davis), injected at 10 mg/kg of body weight. EDTA-anticoagulated blood was collected via peripheral venipuncture. Plasma was separated from whole blood by centrifugation. Either the tissues were fixed in formalin for *in situ* hybridization or mononuclear cells were isolated from the tissues by density gradient centrifugation as described previously (41). DNA extracted from splenocytes was used to screen for the presence of the major histocompatibility complex (MHC) class I alleles Mamu-A*01, -B*01, and -B*08, using a PCR-based technique (58, 59).

### CD4^+^ T cell subpopulation sorting and cell-associated SHIV DNA and RNA quantification.

CD4^+^ T cells were enriched from PBMCs and tissue mononuclear cells using a negative-selection magnetic-activated cell sorting (MACS) system per the manufacturer’s instructions (Miltenyi Biotec, Germany). Enriched CD4^+^ T cells were stained with the fluorescently conjugated antibodies listed in Table S2 in the supplemental material and sorted for naive, memory, and Tfh CD4^+^ T cell subsets (Fig. S5). Total RNA and genomic DNA were isolated using an RNeasy minikit and a DNeasy blood and tissue kit, respectively (Qiagen, Germany). Viral cDNA was generated from the extracted total RNA using SuperScript III reverse transcriptase enzyme (Invitrogen, Carlsbad, CA), PCR nucleotides (New England Biolabs, MA), and a Gag-specific reverse primer (Table S3). The amounts of SHIV DNA and RNA per million CD4^+^ T cells in blood and necropsy tissues were estimated by amplifying cDNA and genomic DNA with the primers and probes described in Table S3, using digital droplet PCR (ddPCR) as described previously (41). The sensitivity of the ddPCR assay was estimated to be detection of 1 SHIV *gag* copy in 10,000 uninfected CD4^+^ T cells. Therefore, an input CD4^+^ T cell count of 10,000 was defined as the threshold cell count (TCC) for the analysis, and samples having input numbers of cells less than the TCC were not analyzed. The amount of SHIV DNA per million CD4^+^ T cells was estimated after normalization of the SHIV *gag* copy numbers with the input CD4^+^ counts. Quantification of SHIV DNA in peripheral lymph node-associated naive, memory, and Tfh CD4^+^ T cell population was performed by quantitative PCR (qPCR) as described previously (21), using the primers and probes described in Table S3.

10.1128/mBio.01971-19.5 FIG S5 Flow cytometry gating strategy for T cell phenotyping and sorting. For T cell phenotyping, CD4^+^ T cells and CD8^+^ T cells were positively selected from the PBMCs by sequential selection of forward and side scatter singlets, lymphocytes, viable cells, CD16^−^ CD14^−^ cells (monocytes/macrophages), and CD3^+^ cells (T cells). CD4^+^ T cells were further analyzed for expression of activation markers HLA-DR and CD69, proliferation marker Ki67, and exhaustion marker PD-1. For sorting of CD4^+^ T cells, CD4^+^ T cells were positively selected from lymph node-associated mononuclear cells by sequential selection of forward-scatter singlets, lymphocytes, and CD3^+^ T cells. Tfh cells (CXCR5^hi^ PD-1^hi^), naive CD4^+^ T cells (CD95^−^ CD28^+^ CD45RA^+^ CCR7^+^), and memory CD4^+^ T cells (CD95^+^ CD28^+^ CD45RA^−^ CCR7^+^) were sorted from CD4^+^ T cells. Download FIG S5, TIF file, 1.9 MB. Copyright © 2019 Goswami et al. 2019 Goswami et al. This is an open-access article distributed under the terms of the Creative Commons Attribution 4.0 International license.

10.1128/mBio.01971-19.7 TABLE S2 Antibodies used for T cell phenotyping, CD4^+^ T cell sorting, and *in situ* hybridization (ISH). Download Table S2, DOCX file, 0.02 MB. Copyright © 2019 Goswami et al. 2019 Goswami et al. This is an open-access article distributed under the terms of the Creative Commons Attribution 4.0 International license.

10.1128/mBio.01971-19.8 TABLE S3 Primers and probes used for the assays. Download Table S3, DOCX file, 0.01 MB. Copyright © 2019 Goswami et al. 2019 Goswami et al. This is an open-access article distributed under the terms of the Creative Commons Attribution 4.0 International license.

### Measurement of HIV Env-specific antibody responses by ELISA.

The plasma concentrations of HIV Env-specific antibodies were estimated by enzyme-linked immunosorbent assay (ELISA), as previously described (33). A human CH22 monoclonal antibody was used as the standard, and the concentration of HIV Env-specific IgG antibody relative to the standard was calculated using a 5-parameter fit curve (SoftMax Pro, version 7, software). The cutoff for positivity for the assay was defined as the optical density (OD) of the rhesus macaque IgG standard with the lowest concentration that was greater than three times the average OD for blank wells. CD4-blocking ELISAs were done as described previously (55).

### Determination of HIV-1 Env-specific IgG epitope specificity and breadth using a binding antibody multiplex assay (BAMA).

HIV antigens were covalently conjugated to polystyrene beads (Bio-Rad), and the binding of IgG to the bead-conjugated HIV-1 antigens in RM plasma samples was measured (33). The antigens used for the assay have been described previously (30). Purified IgG from pooled plasma of HIV-1-vaccinated macaques (RIVIG) was used as a positive control.

### Neutralization assays.

Neutralization of MW965.LucR.T2A.ecto/293T IMC (clade C, tier 1) and the autologous CH505.TF (clade C, tier 2) HIV-1 pseudoviruses by plasma antibodies in TZM-bl cells was measured as previously described (33, 60, 61). The 50% infective dose was calculated as described previously (30). The monoclonal antibody b12R1 was used as a positive control for the MW965 virus, and VRC01 was used a positive control for the CH505 transmitted/founder (T/F) virus.

### ADCC-GTL assay.

An assay with an ADCC-GranToxiLux (GTL) fluorogenic cytotoxicity kit was used to measure plasma ADCC activity as previously described (55, 62). CEM.NKR_CCR5_ target cells were coated with recombinant CH505 gp120. Adult and infant plasma samples were tested after 4-fold serial dilution starting at 1:100.

### SGA.

Single-genome amplification (SGA) of plasma virus was done as described previously (63). The primer used for cDNA preparation was SHIVEnv.R3out. A first round of PCR amplification was conducted using primers SIVmac.F4out and SHIVEnv.R3out A second round of PCR was conducted using primers SIVmac766.F2in and SIVmac766.R2in (Table S3). The *env* gene amplicons obtained were sequenced by Sanger sequencing, and a phylogenetic tree was constructed with the aligned *env* gene sequences by the neighbor-joining method using the SeaView graphical user interface (64). The average pairwise distance was calculated using MEGA (version 6) software (65).

### Tissue-associated infectious viral titers by coculture assay.

Serial dilutions of RM lymphoid and gut-associated mononuclear cells were cocultured with TZM-bl reporter cells as described previously (41) (Fig. S2). The 50% cellular infectious dose (CID_50_) was calculated as the number of mononuclear cells per 10^4^ mononuclear cells required to yield detectable infection of 50% TZM-bl cells, using the method of Reed and Muench (66). The detection threshold of the assay was established to be 2.5 times the mean luminescence output of TZM-bl cells from only 10 independent experiments (876 relative luminescence units [RLU]).

### ISH.

Formalin-fixed, paraffin-embedded tissue sections were seqentially cut (5 μm) and stained for CD3 and CD20 (Table S2) as previously described (67, 68). SHIV RNA was visualized with a 1-plex ViewRNA *in situ* hybridization (ISH) tissue assay kit using SIV_mac239_ or beta-actin (positive control) probe sets and a ViewRNA chromogenic signal amplification kit (Thermo Fisher, Waltham, MA). These two sequential slides were individually imaged with a Zeiss AxioObserver microscope and an AxioCam MRm camera. Composite overlays of CD3/CD20-stained slides with ISH slides were prepared using Zen Lite (version 2.3) software (Zeiss).

### T cell phenotyping.

Phenotyping of rhesus macaque PBMCs and tissue-associated mononuclear cells was performed as described previously (41). The fluorescently conjugated antibodies used to stain the cells are reported in Table S2. For intercellular staining, cells were fixed and permeabilized using an eBioscience FoxP3/transcription factor staining buffer set (Thermo Fisher Scientific) according to the manufacturer’s instructions. The stained cells were acquired on an LSR II flow cytometer (BD Biosciences) using BD FACSDiva software and analyzed with FlowJo (version 10) software (Tree Star, Inc). Gating for all surface and intracellular markers (Fig. S5) was based on the findings for fluorescence-minus-one (FMO) controls. Complete blood counts (CBC) were performed on EDTA-anticoagulated blood samples. Samples were analyzed using a Pentra 60C+ analyzer (ABX Diagnostics). The absolute lymphocyte counts in blood were calculated using the PBMC counts obtained by automated complete blood counting multiplied by the lymphocyte percentages.

### Multiplex analysis of plasma cytokines and chemokines.

Plasma cytokine and chemokine concentrations were assayed in duplicate using a macrophage inflammatory protein 1α (MIP-1α) singleplex kit and a 22-analyte multiplex panel [granulocyte colony-stimulating factor (G-CSF), granulocyte-macrophage colony-stimulating factor (GM-CSF), gamma interferon (IFN-γ), interleukin-1 (IL-1) receptor agonist (IL-1RA), IL-1β, IL-2, IL-4, IL-5, IL-6, IL-8, IL-10, IL-12/23(p40), IL-13, IL-15, IL-17, IL-18, monocyte chemoattractant protein 1 (MCP-1), MIP-1β, sCD40L, transforming growth factor α (TGF-α), tumor necrosis factor alpha (TNF-α), vascular endothelial growth factor (VEGF)] (both from Millipore [catalog number PRCYTOMAG-40K]). The assays were performed according to the manufacturer’s recommended protocol, and the results were read using a FlexMAP three-dimensional array reader (Luminex Corp.). The data were analyzed using Bio-Plex Manager software (Bio-Rad).

### Statistical analysis.

Differences in immune assay measurements between preidentified time points postinfection were performed by the Wilcoxon signed-rank test using the R language and environment for statistical computing (69). Four responses were preselected as primary variables of interest. Additionally, 5 virologic and humoral response-associated variables and 16 T cell phenotype- and plasma inflammatory response-associated variables were selected for exploratory analysis. Cox proportional hazards modeling was used to create univariate survival models for each variable, treating the time to rebound as the event of interest. To account for differences between the adult and neonate groups, a binary indicator for the two different groups was used as a control variable. To identify correlates of viral rebound in infants and adults, Kendall’s tau value between every variable of interest and the time to virus rebound was computed, and the *P* value for the differences in tau values was computed for 10,000 bootstrapped samples. Raw *P* values and/or false discovery rate-adjusted (adjusted by the Benjamini-Hochberg method) *P* values and hazard ratios with confidence intervals are reported for each variable.
