# Supplementary figures and images for: Analytical Treatment Interruption after Short-Term Antiretroviral Therapy in a Postnatally Simian-Human Immunodeficiency Virus-Infected Infant Rhesus Macaque Model
Source: mBio. 2019 Sep 5;10(5):e01971-19. doi: 10.1128/mBio.01971-19 (PMC6945967; doi:10.1128/mBio.01971-19)

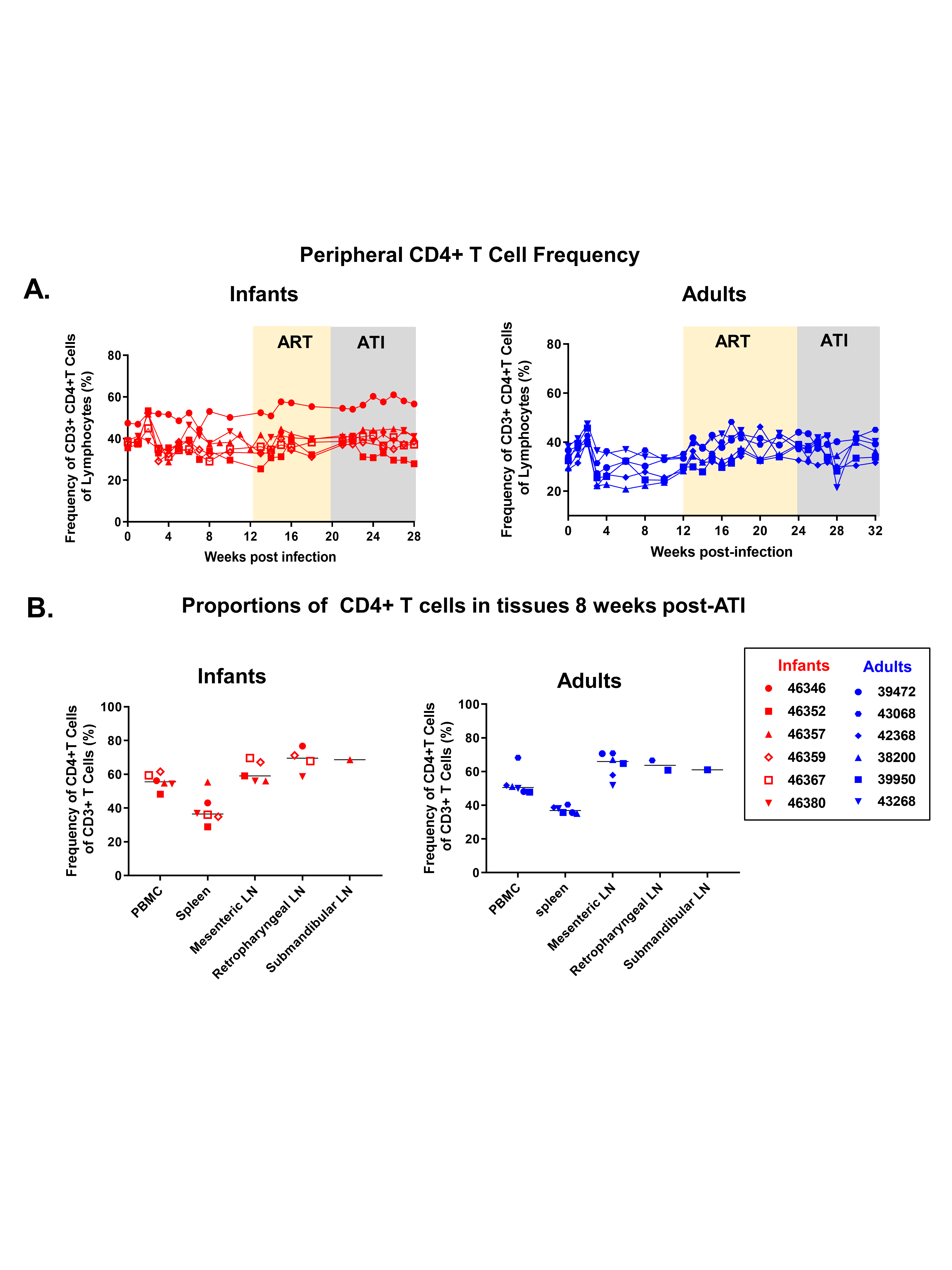

Supplement: FIG S1 [file mBio.01971-19-sf001.tif]

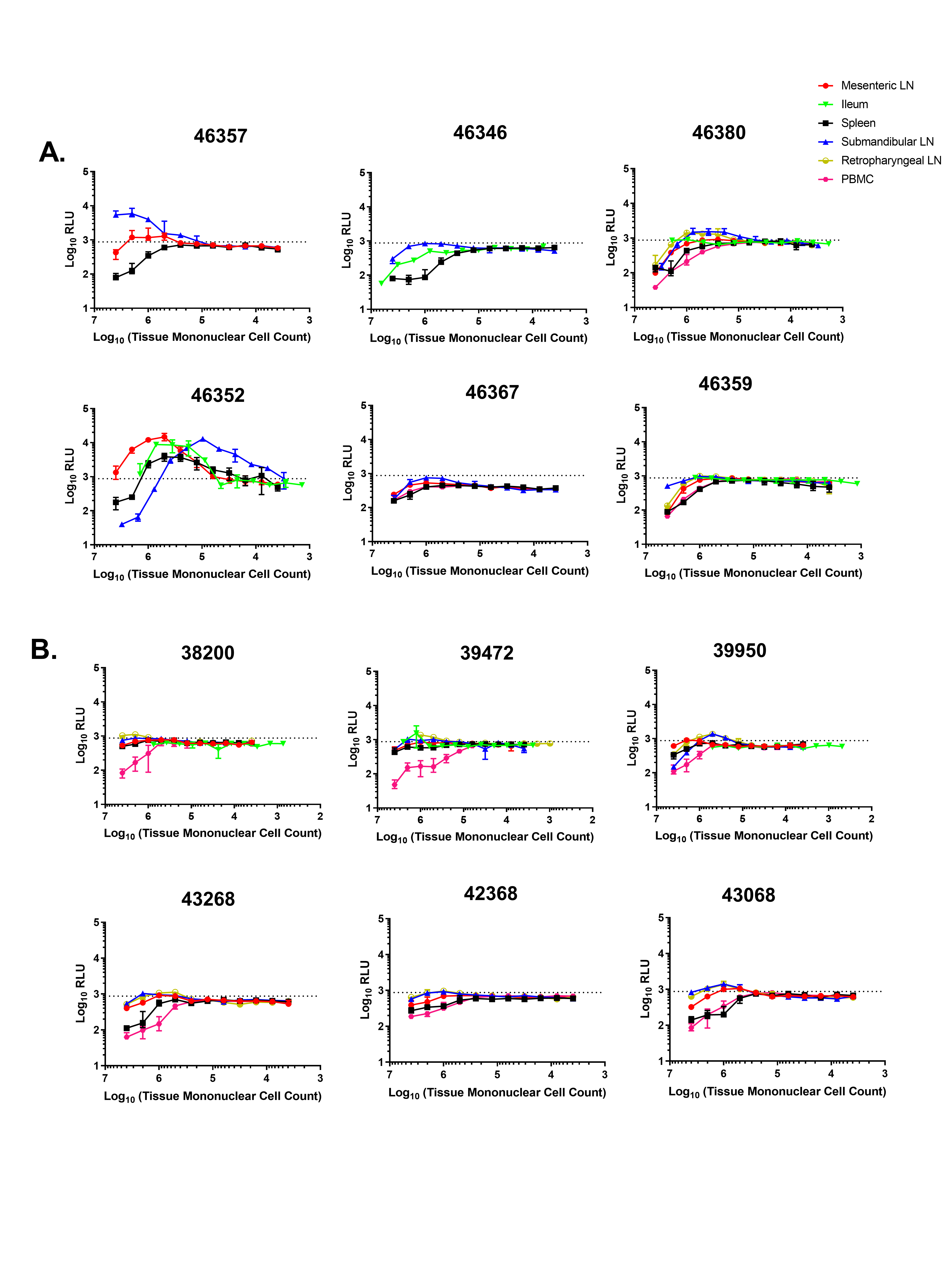

Supplement: FIG S2 [file mBio.01971-19-sf002.tif]

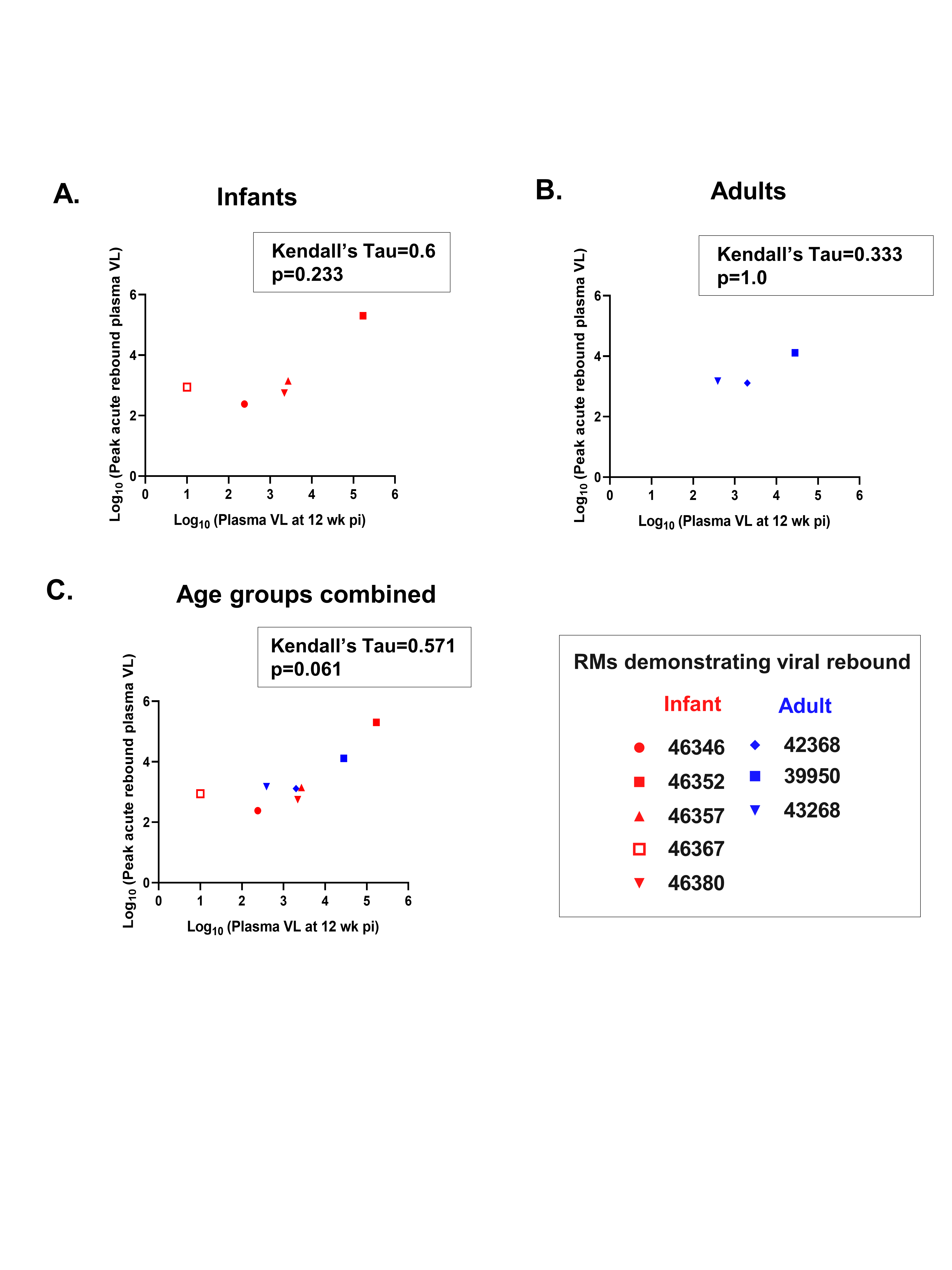

Supplement: FIG S3 [file mBio.01971-19-sf003.tif]

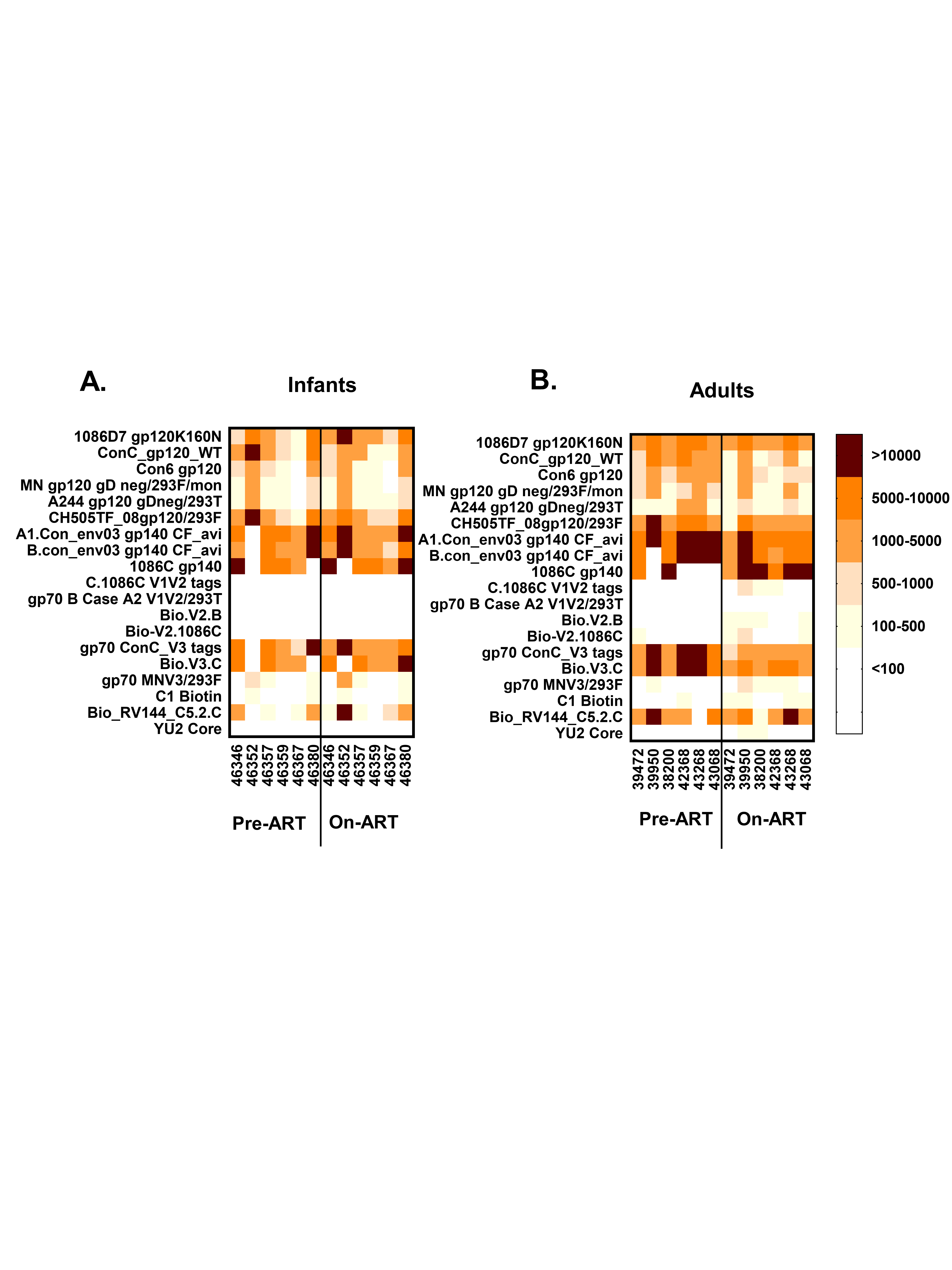

Supplement: FIG S4 [file mBio.01971-19-sf004.tif]

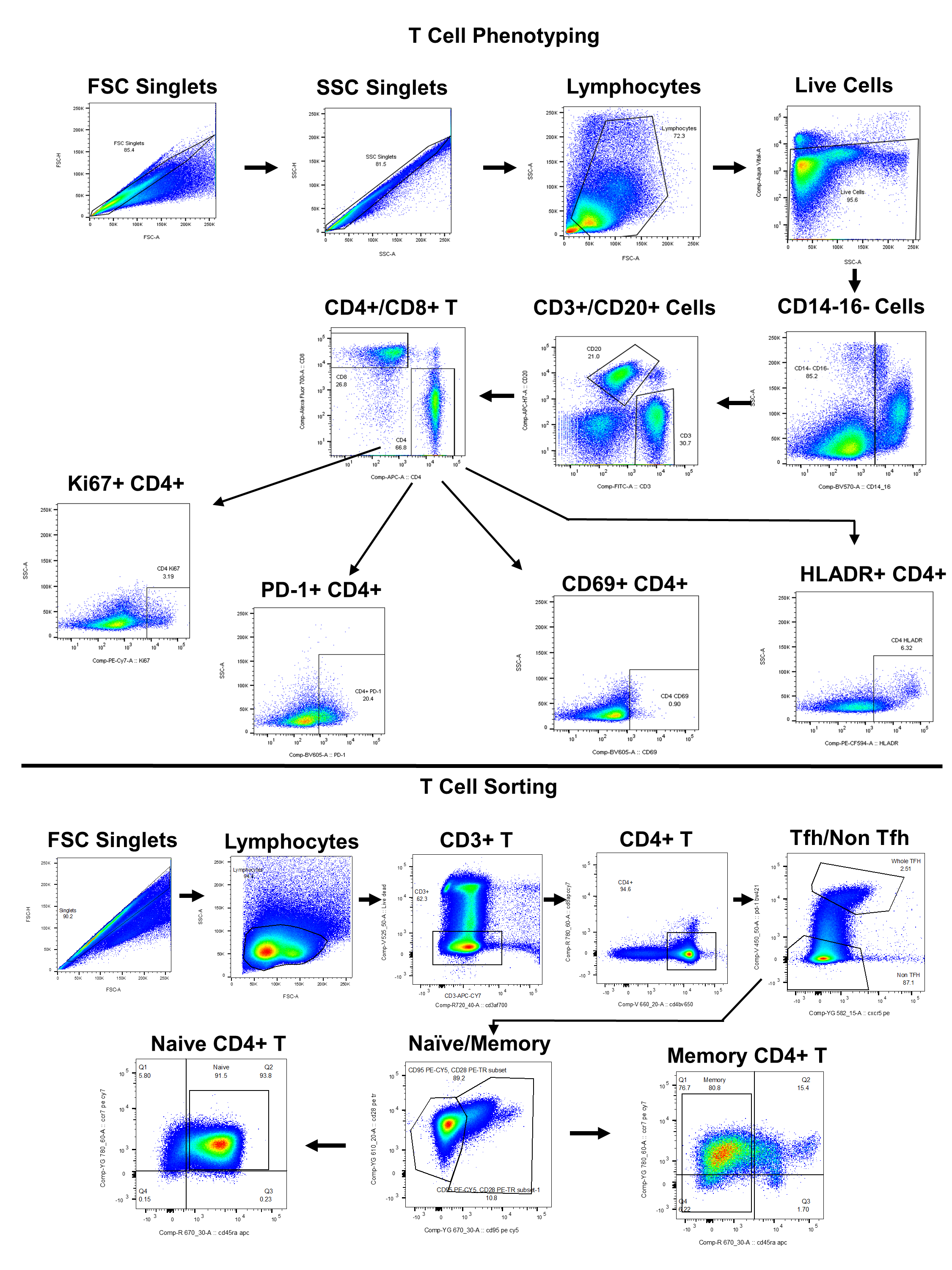

Supplement: FIG S5 [file mBio.01971-19-sf005.tif]
