# Supplementary material for: Analytical Treatment Interruption after Short-Term Antiretroviral Therapy in a Postnatally Simian-Human Immunodeficiency Virus-Infected Infant Rhesus Macaque Model
Source: mBio. 2019 Sep 5;10(5):e01971-19. doi: 10.1128/mBio.01971-19 (PMC6945967; doi:10.1128/mBio.01971-19)
Supplement: TABLE S1 [file mBio.01971-19-st001.docx]

**Table S1.** MHC class I genotype of infant and adult RMs.

|  | **Animal ID** | **MHC Class I^a^** | | | |
| --- | --- | --- | --- | --- | --- |
|  |  | **Mamu A** | |  | **Mamu B** |
|  |  | **A*01** | **A*08** |  | **B*01** |
| Infant | 46357 | - | - |  | +/- |
|  | 46346 | - | - |  | - |
|  | 46352 | - | +/- |  | +/- |
|  | 46359 | - | - |  | - |
|  | 46367 | - | - |  | - |
|  | 46380 | - | - |  | - |
| Adult | 39472 | - | - |  |  |
|  | 43068 | - | - |  | +/- |
|  | 43268 | - | - |  | +/- |
|  | 42368 | - | - |  | - |
|  | 39950 | - | - |  | - |
|  | 38200 | - | - |  | +/+ |

^a^Mamu A*01 has been associated with attenuation of SHIV disease progression and Mamu B*01 has been associated with better SIV disease progression.

+/- indicate heterozygous.
