# Supplementary material for: Analytical Treatment Interruption after Short-Term Antiretroviral Therapy in a Postnatally Simian-Human Immunodeficiency Virus-Infected Infant Rhesus Macaque Model
Source: mBio. 2019 Sep 5;10(5):e01971-19. doi: 10.1128/mBio.01971-19 (PMC6945967; doi:10.1128/mBio.01971-19)
Supplement: TABLE S2 [file mBio.01971-19-st002.docx]

**Table S2.** Antibodies used for T cell phenotyping, CD4+ T cell sorting and *in situ* hybridization (ISH).

|  | **Marker** | **Fluorophore** | **Staining type** | **Clone** | **Vendor** | **Secondary Antibody** |
| --- | --- | --- | --- | --- | --- | --- |
| **T Cell Phenotyping** | CD20 | APC-H7 | Surface | 2H7 | BD Biosciences | N/A |
|  | CD3 | FITC | Surface | SP34 | BD Biosciences | N/A |
|  | CD4 | APC | Surface | L200 | BD Biosciences | N/A |
|  | CD8 | AF700 | Surface | SK1 | BD Biosciences | N/A |
|  | Ki67 | PEcy7 | Intracellular | B56 | BD Biosciences | N/A |
|  | HLADR | PE-CF594 | Surface | G46-6 | BD Biosciences | N/A |
|  | CD69 | BV605 | Surface | FN50 | BD Biosciences | N/A |
|  | CD14 | BV570 | Surface | M5E2 | Biolegend | N/A |
|  | CD16 | BV570 | Surface | 3G8 | Biolegend | N/A |
|  | PD-1 | BV605 | Surface | EH12.2H7 | Biolegend | N/A |
| **CD4+ T Cell Sorting** | CD3 | AF700 | Surface | SP34-2 | BD Biosciences | N/A |
|  | CCR7 | PE-CY7 | Surface | CD197 | BD Biosciences | N/A |
|  | CD8 | APC-CY7 | Surface | SK1 | BD Biosciences | N/A |
|  | CD45RA | APC | Surface | 5H9 | BD Biosciences | N/A |
|  | CD95 | PE-CY5 | Surface | DX2 | BD Biosciences | N/A |
|  | CD28 | ECD | Surface | CD28.2 | Beckman Coulter | N/A |
|  | CD4 | BV650 | Surface | OKT4 | Biolegend | N/A |
|  | PD-1 | BV421 | Surface | EH12.2H7 | Biolegend | N/A |
|  | CXCR5 | PE | Surface | MU5UBEE | eBiosciences | N/A |
| **ISH** | CD3 | None | Surface | Polyclonal rabbit IgG | Dako | Goat anti-rabbit AF488 (Invitrogen) |
|  | CD20 | None | Surface | Mouse IgG2a-L26 | Dako | Goat anti-mouse AF594 (Invitrogen) |

^a^N/A, Not applicable
