# Supplementary material for: Analytical Treatment Interruption after Short-Term Antiretroviral Therapy in a Postnatally Simian-Human Immunodeficiency Virus-Infected Infant Rhesus Macaque Model
Source: mBio. 2019 Sep 5;10(5):e01971-19. doi: 10.1128/mBio.01971-19 (PMC6945967; doi:10.1128/mBio.01971-19)
Supplement: TABLE S3 [file mBio.01971-19-st003.docx]

**Table S3.** Primers and probes used for the assays.

| **Primers and Probes** | **Identifier** | **Type** | **Assay** |
| --- | --- | --- | --- |
| 5’-CTACTGGTCTCCTCCAAAGAGAGAATTG-3’ | Gag-specific reverse primer | Primer | cDNA production |
| 5’-TCAGGCACTGTCAGAAGGTT-3’ | Gag-specific forward primer | Primer | ddPCR |
| 5’ TTGTTGTGGAGCTGGTTGTG-3’ | Gag-specific reverse primer | Primer | ddPCR |
| 5’-FAM-AGCCGCTTGATGGTCTCCCACA-TAMRA-3’ | SHIV CH505 gag-specific probe | Probe | ddPCR |
| 5’ -GAAGGTGAAGGTCGGAGTC-3’ | Gag-specific forward primer | Primer | qPCR |
| 5’-GAAGATGGTGATGGGATTTC-3’ | Gag-specific reverse primer | Primer | qPCR |
| 5’ -TGCATGAGAAAACGCCAGTAA-3’ | Albumin-specific forward primer | Primer | qPCR |
| 5’-ATGGTCGCCTGTTCACCAA-3’ | Albumin-specific reverse primer | Primer | qPCR |
| 5’-FAM CAAGCTTCCCGTTCTCAGCC TAMRA-3’ | SIVmac gag-specific probe | Probe | qPCR |
| 5’-6-FAM AGAAAGTCACCAAATGCTGCACGGAATC-3’-6-TAMRASp | Albumin probe | Probe | qPCR |
| 5’-CTAATTCCTGGTCCTGAGGTGTAATCCTG-3’ | SHIVEnv.R3out | Primer | SGA |
| 5’-TCATATCTATAATAGACATGGAGACACCC-3’ | SIVmac.F4out | Primer | SGA |
| 5’- CTAATTCCTGGTCCTGAGGTGTAATCCTG-3’ | SHIVEnv.R3out | Primer | SGA |
| 5’- GGAAATCCTCTCTCAACTATACCGCCCTC -3’ | SIVmac766.F2in | Primer | SGA |
| 5’- CTATTGCCAATTTGTAACTCATTGTTC-3’ | SIVmac766.R2in | Primer | SGA |
